# Socio-protective effects of active case finding on catastrophic costs from tuberculosis in Ho Chi Minh City, Viet Nam: a longitudinal patient cost survey

**DOI:** 10.1186/s12913-021-06984-2

**Published:** 2021-10-05

**Authors:** Luan Nguyen Quang Vo, Rachel Jeanette Forse, Andrew James Codlin, Ha Minh Dang, Vinh Van Truong, Lan Huu Nguyen, Hoa Binh Nguyen, Nhung Viet Nguyen, Kristi Sidney-Annerstedt, Knut Lonnroth, S Bertel Squire, Maxine Caws, Eve Worrall, Noemia Teixeira de Siqueira-Filha

**Affiliations:** 1 Friends for International TB Relief, 1/21 Le Van Luong, Nhan Chinh, Thanh Xuan, Ha Noi, Vietnam; 2 IRD VN, Ho Chi Minh City, Vietnam; 3 Department of Global Public Health, Karolinska Institutet, Solna, Sweden; 4 Pham Ngoc Thach Hospital, Ho Chi Minh City, Vietnam; 5 National Lung Hospital, Ha Noi, Vietnam; 6 Liverpool School of Tropical Medicine, Department of Clinical Sciences, Liverpool, UK; 7 Birat Nepal Medical Trust, Lazimpat, Kathmandu Nepal; 8 Department of Health Sciences, University of York, York, UK

**Keywords:** Tuberculosis, Catastrophic costs, Active case finding, Social protection, Patient cost survey, Longitudinal design, Viet Nam

## Abstract

**Background:**

Many tuberculosis (TB) patients incur catastrophic costs. Active case finding (ACF) may have socio-protective properties that could contribute to the WHO End TB Strategy target of zero TB-affected families suffering catastrophic costs, but available evidence remains limited. This study measured catastrophic cost incurrence and socioeconomic impact of an episode of TB and compared those socioeconomic burdens in patients detected by ACF versus passive case finding (PCF).

**Methods:**

This cross-sectional study fielded a longitudinal adaptation of the WHO TB patient cost survey alongside an ACF intervention from March 2018 to March 2019. The study was conducted in six intervention (ACF) districts and six comparison (PCF) districts of Ho Chi Minh City, Viet Nam. Fifty-two TB patients detected through ACF and 46 TB patients in the PCF cohort were surveyed within two weeks of treatment initiation, at the end of the intensive phase of treatment, and after treatment concluded. The survey measured income, direct and indirect costs, and socioeconomic impact based on which we calculated catastrophic cost as the primary outcome. Local currency was converted into US$ using the average exchange rates reported by OANDA for the study period (VNĐ1 = US$0.0000436, 2018–2019). We fitted logistic regressions for comparisons between the ACF and PCF cohorts as the primary exposures and used generalized estimating equations to adjust for autocorrelation.

**Results:**

ACF patients were poorer than PCF patients (multidimensional poverty ratio: 16 % vs. 7 %; *p* = 0.033), but incurred lower median pre-treatment costs (US$18 vs. US$80; *p* < 0.001) and lower median total costs (US$279 vs. US$894; *p* < 0.001). Fewer ACF patients incurred catastrophic costs (15 % vs. 30 %) and had lower odds of catastrophic cost (aOR = 0.17; 95 % CI: [0.05, 0.67]; *p* = 0.011), especially during the intensive phase (OR = 0.32; 95 % CI: [0.12, 0.90]; *p* = 0.030). ACF patient experienced less social exclusion (OR = 0.41; 95 % CI: [0.18, 0.91]; *p* = 0.030), but more often resorted to financial coping mechanisms (OR = 5.12; 95 % CI: [1.73, 15.14]; *p* = 0.003).

**Conclusions:**

ACF can be effective in reaching vulnerable populations and mitigating the socioeconomic burden of TB, and can contribute to achieving the WHO End TB Strategy goals. Nevertheless, as TB remains a catastrophic life event, social protection efforts must extend beyond ACF.

**Supplementary Information:**

The online version contains supplementary material available at 10.1186/s12913-021-06984-2.

## Background

Tuberculosis (TB) disproportionally affects the poorest segments of society [1]. Economically and socially vulnerable persons are at higher risk of TB infection and progression to active TB disease [2–4]. Previous research has also shown that TB exacerbates poverty [5–7]. Thus, one of the three targets included in the WHO End TB Strategy is the elimination of catastrophic costs, defined as incurring expenses in excess of 20 % of annual household income, due to TB [8].

Despite recent rapid economic development, Viet Nam remains a high TB burden country. There were 170,000 incident cases and 11,400 TB-related mortalities in 2019 [9]. Viet Nam conducted its first national TB patient cost survey in 2016 [10]. This cross-sectional survey sampled 677 drug-susceptible (DS-TB) and 58 multi-drug resistant TB (MDR-TB) patients. The proportion of DS-TB patients who experienced catastrophic costs was 63 %, while the catastrophic costs prevalence among MDR-TB patients was 98 % [11]. Following the national survey, the National TB Control Program (NTP) explored and developed various social protection mechanisms for TB patients. Most notable is the establishment of a national patient support fund named the Patient Support Foundation to End Tuberculosis to pay for Social Health Insurance (SHI) enrollment and unreimbursed medical costs.

With a treatment coverage rate of 60 %, there remains a pool of undetected TB and sustained transmission in the community [9]. Active case finding (ACF) is a strategy to find people with TB earlier in their disease course and in larger numbers, particularly among vulnerable populations [12]. If implemented consistently, ACF has the potential to reduce TB transmission and improve health outcomes over time [13]. Based on these epidemiologic benefits, the NTP has placed a strong emphasis on ACF in the recent past [14–17]. However, to date the evidence on the socio-protective nature of ACF remains limited [18, 19] with none available for the Vietnamese context.

This study aimed to assess the socioeconomic impact and socio-protective potential of ACF by obtaining a longitudinal measure of the financial burden of DS-TB treatment and comparing costs in patients detected through active versus passive case finding.

## Methods

### Study design

This cross-sectional study fielded a longitudinal cost survey from March 2018 to March 2019 alongside an intervention. The intervention consisted of ACF among household and close contacts, and persons living in presumptive hotspots, boarding homes and urban slums implemented by community health workers (CHW). All household contacts and other targeted persons with symptoms suggestive of TB were referred for chest x-ray screening and sputum testing by rapid molecular assay or smear microscopy. The CHWs supported patients to initiate and adhere to treatment. Patients in the PCF districts received routine care as per national treatment guidelines. The intervention is described in detail elsewhere [20, 21].

### Study setting

**Fig. 1:**
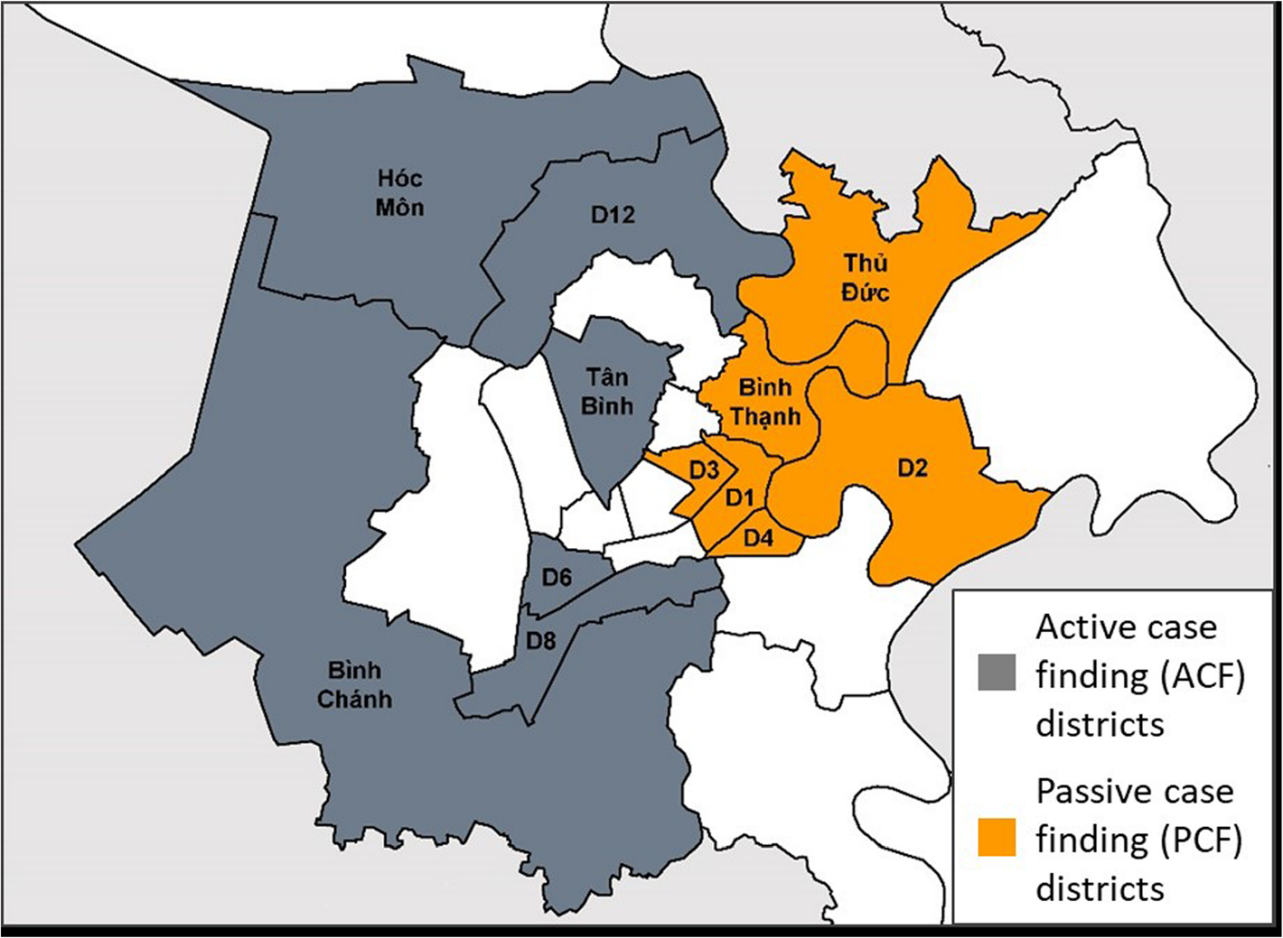
Location of sampling districts in Ho Chi Minh City, Viet Nam

The study took place in 12 districts of Ho Chi Minh City (HCMC), Viet Nam (Fig. 1). Six districts implemented a community-based ACF intervention. The remaining six districts served as control areas where people with TB were passively detected through routine program activities (PCF). In 2017, the intervention area had a population of 2,814,034 and notified 4,159 TB patients, while the control area had a population of 1,789,396 and notified 2,859 TB patients [22]. This represented a case notification rate (CNR) of 148 per 100,000 and 160 per 100,000, respectively.

### Participant recruitment and sample size

The study employed a consecutive sampling approach in both cohorts. Patients entered into TB treatment registers were referred by District TB Unit staff for recruitment to participate on the study. We included pulmonary DS-TB patients 18 years and older who resided in the study area. We excluded persons treated in the private sector or declined to participate.

We estimated a sample size of 100 patients equally distributed between ACF and PCF cohorts. The sample size was based on a 63 % catastrophic cost incurrence rate among DS-TB patients [11]. We estimated an 80 % power to show a 50 % reduction in catastrophic costs from ACF [12] at 95 % confidence, and with 25 % contingency for loss to follow-up.

### Data collection

We developed a longitudinal adaptation of the WHO survey instrument used in Viet Nam’s national patient cost survey to measure patient cost and socioeconomic impact throughout TB treatment and limit recall bias (supplementary material) [23]. We expanded the survey’s asset list with items relevant in the contemporary urban Vietnamese context. The questionnaire assessed participant characteristics, financial and economic costs, and socioeconomic impact associated with TB. The latter included changes in employment status, food insecurity, productivity loss, social exclusion and use of coping strategies. Clinical data such as TB diagnosis and treatment information were extracted from patient registers.

**Fig. 2:**
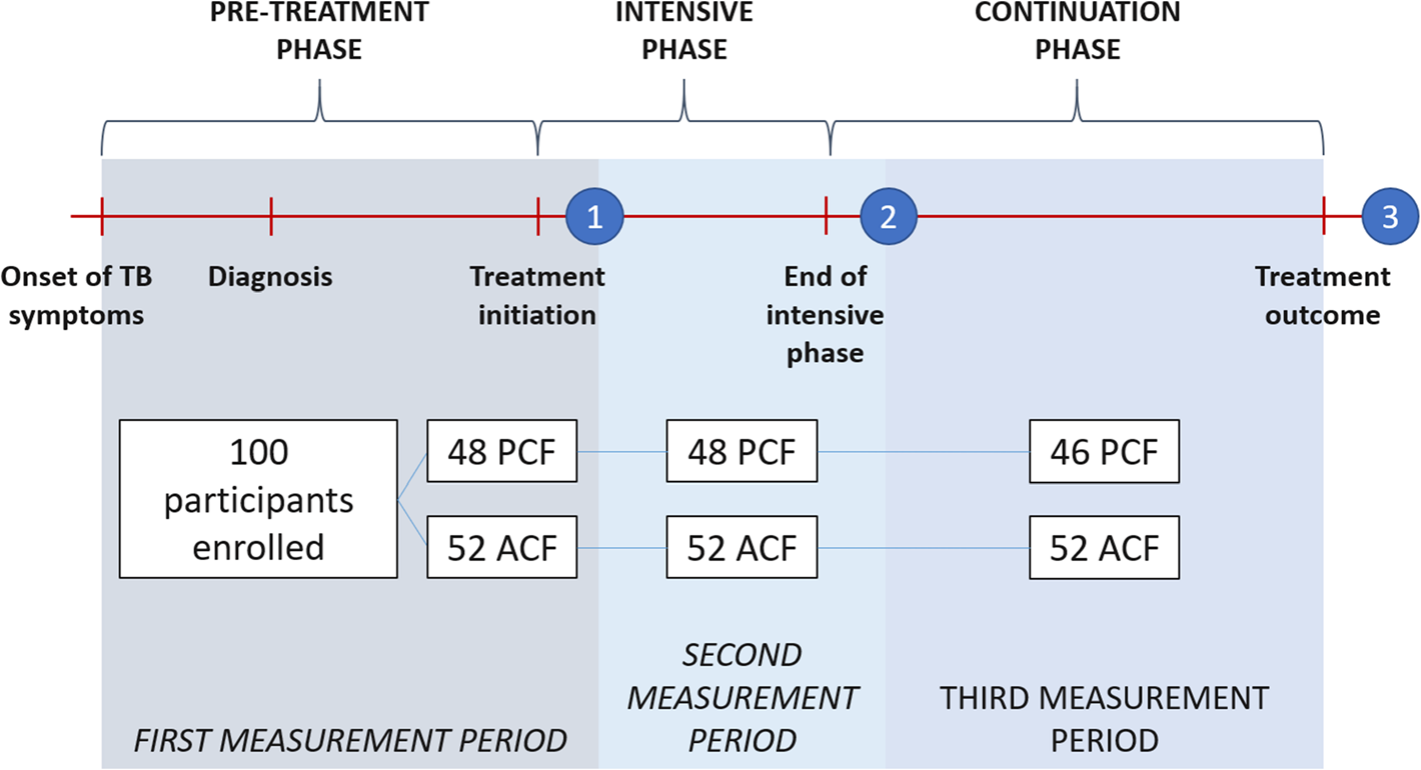
Survey timeline and participant retention

In Viet Nam, new and relapse DS-TB patients typically take treatment for six months. The intensive phase lasts two months and the continuation phase lasts four months. Costs and social impact were measured thrice in both ACF and PCF cohorts to cover the pre-treatment, intensive and continuation phases. The first interview took place after at least two weeks of treatment and covered the period from the onset of symptoms to the time of the interview. The second and third interviews occurred after completion of the intensive and continuation phases, respectively (Fig. 2).

Interviewers captured responses through audio recordings and on paper. Data were digitized using web-forms on the ONA platform (ONA Systems, Nairobi, Kenya); digital data were checked against the paper surveys to ensure data entry accuracy. All costs were collected in Viet Nam Dong (VNĐ) and converted to US dollars (US$) using the average 2018–2019 exchange rates reported by OANDA for the study period (VNĐ1 = US$0.0000436) [24].

### Data analysis

#### Participant characteristics and health-seeking behaviors

 We presented participant demographic, clinical and socioeconomic characteristics. We employed an adapted multidimensional poverty index for TB (MPI-TB) using the Alkire-Foster method to profile a participant’s socioeconomic status [25–27]. The MPI-TB construct was designed as an absolute measure with thresholds mirroring those of Viet Nam’s national MPI, and is further detailed in the supplementary material. Health-seeking behavior was summarized by frequency and type of healthcare provider visited.

#### Patient income and costs

For each treatment period, we collected monthly personal and household income and estimated direct medical, direct non-medical and indirect costs incurred. Direct medical costs included medications, diagnostic tests, consultations and hospitalization fees. Direct non-medical costs included spend on transportation and food. Indirect cost consisted of income loss, which was self-reported by the patient. For those patients who could not provide this information, we calculated income loss by using the self-reported number of working hours/days lost multiplied by the hourly (US$0.90) or daily (US$7.00) minimum wage for Region 1 in Viet Nam [28]. Indirect costs, time lost traveling to health facilities and waiting for an appointment were calculated by multiplying the number of hours lost by the hourly minimum wage. Total indirect cost was calculated by summing the monetary value of the time and income lost. We excluded caregiver time-loss for consistency with the national TB patient cost survey [11] and patients lost to follow-up after the first interview.

#### Socioeconomic impact

We reported the frequency of financial coping mechanisms used, loss of employment, reduction in work hours, lower wage levels or the transfer to a lower paying position or job. We estimated ratios of catastrophic costs and poverty headcount. To estimate catastrophic cost, we applied the WHO definition [10] of total costs exceeding 20 % of the average annual household income before this episode of TB. We applied the poverty headcount defined by the World Bank as US$1.90 per day with an annual purchasing power parity conversion factor for Gross Domestic Product at 2017 prices as defined by the World Bank [29, 30].

### Statistical approach

Chi-square and Fisher’s exact tests were used to determine statistical differences in proportions of categorical variables. Wilcoxon rank-sum tests were used to compare median costs between the ACF and PCF cohorts. We excluded missing data and reported denominators that deviated from the total sample size. Univariate and multivariate logistic models were fitted to measure the association between catastrophic costs incurrence and ACF/PCF cohort alongside other demographic and socioeconomic patient characteristics. Differences in socioeconomic impact and catastrophic costs in individual treatment periods were analyzed by fitting univariate population-averaged logistic regression models using generalized estimation equation methods with working correlation structures based on Quasi-likelihood Information Criteria. Hypothesis tests were two-sided. P-values below 0.05 were considered statistically significant. Data analysis was performed in Stata v15 (StataCorp, College Station, TX, USA).

### Ethical considerations

Approvals were granted by the Liverpool School of Tropical Medicine Research Ethics Committee (17–019) and Pham Ngoc Thach Provincial TB Hospital Institutional Review Board (430/HDDD-PNT). The implementation of the interventions was approved by the HCMC People’s Committee (214/QD-UBND, 2138/QD-UBND, 2878/QD-UBND). The NTP approved use of programmatic treatment data. All protocols were carried out in accordance with relevant guidelines and regulations. Before the interview, we provided a written participant information sheet, a verbal explanations of the study and an opportunity to ask questions to ensure all participants were aware of the study. All participants provided informed written consent. All data were anonymized prior to analysis.

## Results

### Participant characteristics

One-hundred invited individuals agreed to participate in the survey, but two did not complete the final interview. Thus, the final study sample consisted of 98 participants, 46 PCF and 52 ACF, for a total of 294 survey encounters. Most patients were male (62/98 = 63 %) (Table 1). ACF patients tended to be slightly older than PCF patients (52 vs. 45; p = 0.063). The ACF cohort had fewer individuals with a secondary school degree (54 % vs. 78 %; p = 0.011) and more individuals whose income fell in the poorest tertile (43 % vs. 22 %; p = 0.003). The share of people enrolled onto SHI in the ACF cohort was significantly lower than in the PCF cohort (69 % vs. 91 %; p = 0.007). The depth-adjusted MPI-TB ratio among ACF patients was significantly higher than among PCF patients (15.5 % vs. 7.1 %; p = 0.033).

**Table 1:** Baseline socioeconomic and treatment characteristics of TB patients in the ACF and PCF cohorts

	ACF (*N* = 52)	PCF (*N* = 46)	All (*N* = 98)	*P*-value^g^
**Socioeconomic characteristics**				
Male, N (%)	33 (63)	29 (63)	62 (63)	0.966
Age, mean (SD), years	51.8 (15.7)	44.8 (17.9)	48.5 (17.0)	0.063
Complete secondary school, N (%)	28 (54)	36 (78)	64 (65)	0.011*
Employed before the episode of TB, N (%)	39 (75)	33 (72)	72 (73)	0.715
*Patient income pre-TB, N (%)*^a^				
Poorest	22 (43)	10 (22)	32 (33)	0.003*
Moderate	18 (35)	14 (31)	32 (33)	0.714
Wealthiest	13 (25)	19 (42)	32 (33)	0.072
*Household income pre-TB, N (%)*^a^				
Poorest	21 (40)	12 (27)	33 (34)	0.155
Moderate	18 (35)	14 (31)	32 (33)	0.714
Wealthiest	13 (25)	19 (42)	32 (33)	0.072
Social Health Insurance, N (%)	36 (69)	42 (91)	78 (80)	0.007*
Adjusted MPI-TB headcount ratio, mean (SD)^b^	15.5 (21.8)	7.1 (15.9)	11.6 (19.6)	0.033*
**TB diagnosis and treatment**				
TB/HIV co-infection, N (%)	0 (0)	3 (7)	3 (3)	0.061
New TB case, N (%)	35 (67)	44 (96)	79 (81)	< 0.001*
Bacteriologically confirmed, N (%)	52 (100)	43 (93)	95 (97)	0.061
Time between onset of TB symptoms and treatment initiation^c^, mean (SD), weeks	9.1 (8.9)	12.7 (18.2)	11.0 (14.5)	0.245
Hospitalization pre-treatment, N (%)	3 (6)	10 (22)	13 (13)	0.020*
Hospitalization during treatment, N (%)	3 (6)	6 (13)	9 (9)	0.213
Number of follow-up visits during treatment, mean (SD)	3.3 (1.0)	3.5 (1.0)	3.4 (1.0)	0.402
*Type of service visited, pre-treatment, N (%)*^*d*^				
Private sector	31/262 (12)	79/366 (22)	110/628 (18)	0.002*
Non-NTP public sector	34/262 (13)	140/366 (38)	174/628 (28)	< 0.001*
NTP^e^	110/262 (42)	63/366 (17)	173/628 (28)	< 0.001*
Others^f^	87/262 (33)	84/366 (23)	171/628 (27)	< 0.004*
*Type of service visited, treatment, N (%)*^*d*^				
Private sector	8/234 (3)	25/273 (9)	33/507 (7)	0.009*
Non-NTP public sector	75/234 (32)	57/273 (21)	132/507 (26)	0.004*
NTP^e^	2/234 (1)	40/273 (15)	42/507 (8)	< 0.001*
Others^f^	149/234 (64)	151/273 (55)	300/507 (59)	0.056

^a^Based on within-sample tertiles of self-reported incomes;

^b^Headcount ratio calculated based on a deprivation threshold of 33 % (> 5 deprivations) as adapted from official guidelines on multi-dimensional poverty defined by the Government of Viet Nam with further detail provided in the supplementary material;

^c^Contains nine missing values in the ACF cohort with *N* = 43;

^d^Total number of visits during the pre-treatment (ACF = 262, PCF = 366, Total = 628) and treatment periods (ACF = 234, PCF = 273, Total = 507);

^e^*NTP *National TB Control program;

^f^Traditional healers, herbalists, pharmacists and mobile X-ray events (pre-treatment, only);

^g^Chi-square, Fischer Exact (any cell with n < 5 in the contingency table) for proportions, Wilcoxon Rank Sum for medians, t-test for means

* Statistically significant difference at 95 % confidence level

### Health-seeking behaviors

There were fewer new TB patients in the ACF cohort (67 % vs. 96 %; p < 0.001) and fewer pre-treatment hospitalizations (6 % vs. 22 %; p = 0.003) than among PCF patients. ACF patients reported fewer visits to health facilities before initiating treatment (262 vs. 366) with a significantly higher share of ACF patients accessing NTP services compared PCF patients (42 % vs. 17 %; p < 0.001). Conversely, the rate of health-seeking in non-NTP public healthcare facilities (13 % vs. 38 %; p < 0.001) and private healthcare providers (12 % vs. 22 %; p = 0.002) was significantly lower in ACF patients.

### Patient income and costs

**Fig. 3:**
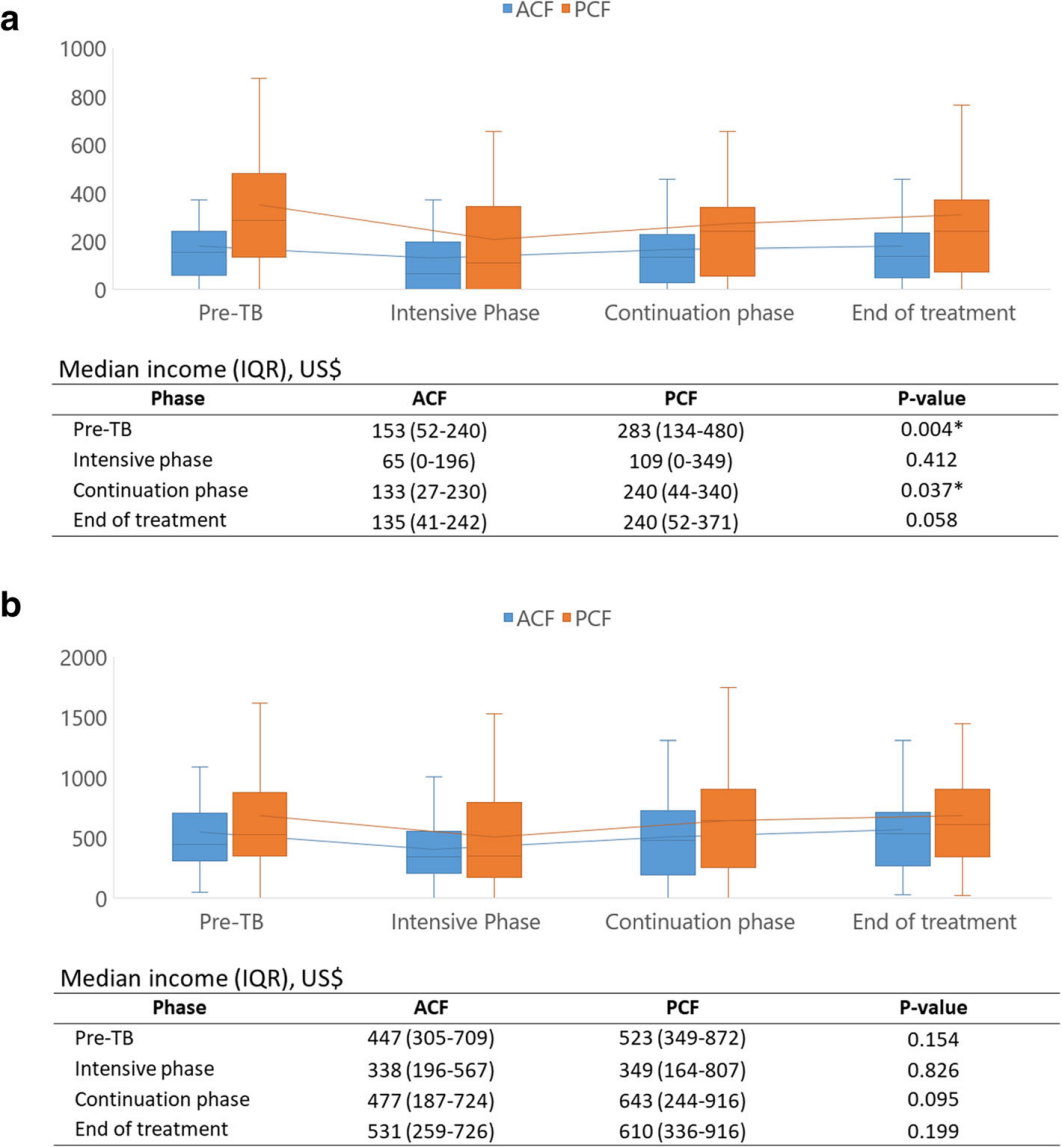
Variation in income by treatment period and study cohort. **a**) Personal income. **b**) Household income

Prior to TB treatment, the monthly median personal income was significantly lower in the ACF cohort compared to the PCF cohort in the pre-treatment (US$153 vs. US$283; p = 0.004) and continuation phase (US$133 vs. US$240; p = 0.037) (Fig. 3a). During the intensive phase the median personal income declined sharply from US$153 to US$65 in the ACF cohort and from US$283 to US$109 in the PCF cohort, but recovered to pre-TB levels by the end of treatment. Median household income was lower in the ACF cohort than in the PCF cohort in all periods, but these differences were not significant. The household income changes followed the same pattern as observed on personal income, declining during the intensive phase and recovering by the end of the treatment (Fig. 3b).

Table 2 shows the mean and median costs incurred during the pre-treatment and treatment periods as well as the total costs and income losses incurred due to the episode of TB. The median total costs incurred by ACF patients during the pre-treatment period was significantly lower compared to PCF patients (US$18 vs. US$80; p < 0.001). Specifically, patients in the ACF cohort incurred significantly lower direct medical costs compared to the PCF cohort (US$13 vs. US$75;, p < 0.001). Once TB treatment began, there was no significant difference in direct, indirect or total costs between the ACF and PCF cohorts.

**Table 2:** Mean and median direct and indirect costs during pre-treatment and treatment periods for TB patients in the ACF and PCF cohorts (VNĐ1 = US$0.0000436, 2018-2019)

**Cost item**	**ACF (*N* = 52)**		**PCF (*N*** **= 46)**		**All (*N*** **= 98)**		***P*-value**^a^
	**Mean (95% CI)**	**Median (IQR)**	**Mean (95% CI)**	**Median (IQR)**	**Mean (95% CI)**	**Median (IQR)**	
**Pre-treatment **							
Direct medical	37 (18-56)	13 (4-31)	151 (70-231)	75 (30-168)	90 (50-130)	29 (11-81)	<0.001*
Direct non-medical	7 (2-12)	2 (0-4)	6 (3-9)	3 (1-8)	7 (4-9)	2 (1-6)	0.1394
*Total direct*^b^	*44 (22-66)*	*17 (6-34)*	*155 (73-238)*	*78 (32-154)*	*96 (55-137)*	*34 (11-88)*	<0.001*
Indirect, time loss	5 (0-10)	1 (0.5-1)	20 (3-36)	2 (1-5)	12 (4-20)	1 (1-3)	<0.001*
*Total pre-treatment*	*49 (24-74)*	*18 (7-37)*	*175 (85-264)*	*80 (33-203)*	*108 (63-153)*	*35 (13-90)*	<0.001*
**Treatment **							
Direct medical	54 (39-70)	34 (22-61)	384 (76-693)	76 (37-154)	209 (63-355)	47 (24-93)	<0.001*
Direct non-medical	44 (31-57)	27 (12-59)	43 (14-71)	14 (8-38)	43 (29-58)	20 (10-47)	0.056
*Total direct*^b^	*98 (74-122)*	*66 (43-121)*	*427 (98-756)*	*90 (50-195)*	*253 (97-408)*	*83 (46-160)*	*0.067*
Indirect, time loss	13 (7-20)	6 (4-13)	27 (1-53)	5 (4-8)	20 (7-33)	5 (4-11)	0.144
*Total treatment*	*112 (85-139)*	*73 (47-143)*	*454 (105-804)*	*95 (55-202)*	*273 (108-438)*	*91 (50-174)*	*0.109*
**Total costs (pre-treatment + treatment)**							
Direct medical	91 (68-115)	62 (34-123)	535 (169-902)	166 (98-289)	230 (125-474)	105 (47-196)	<0.001*
Direct non-medical	51 (37-65)	34 (14-73)	48 (19-79)	19 (13-44)	50 (34-65)	23 (13-59)	0.095
*Total Direct *	*142 (84-139)*	*106 (72-200)*	*582 (205-925)*	*179 (99-351)*	*349 (153-505)*	*131 (84-258)*	0.001*
Income loss	273 (145-400)	71 (0-272)	627 (384-869)	414 (0-931)	439 (304-573)	134 (0-761)	0.032*
Time loss	18 (10-27)	8 (5-15)	47 (11-83)	9 (6-23)	32 (14-49)	8 (5-19)	0.693
*Total Indirect*	*291 (163.7-419.0)*	*115 (12-307)*	*674 (436-912)*	*460 (59-938)*	*471 (337-605)*	*166 (23-765)*	0.003*
*Total costs*	*434 (301-566)*	*279 (122-468)*	*1,256 (807-1,706)*	*894 (234-1359)*	*820 (587-1,053)*	*413 (168-1,046)*	<0.001*

^a^Wilcoxon Rank Sum;

^b^Net value: reimbursement deducted

* Statistically significant difference at 95 % confidence level

Overall, ACF patients incurred significantly fewer total costs than PCF patients (US$279 vs. US$894; p < 0.001), and significantly lower indirect costs (US$115 vs. US$460; p = 0.003) and income loss (US$71 vs. US$414; p = 0.032).

### Socioeconomic impact

Fewer ACF patients incurred catastrophic costs than PCF patients (15 % vs. 30 %) and ACF patients had significantly lower odds of catastrophic cost (aOR = 0.17; 95 % CI: [0.05, 0.67]; p = 0.011) (Table 3). This lower rate of catastrophic cost incurrence in the ACF cohort was evident across all individual treatment periods (Table 4). Particularly during the intensive phase, catastrophic cost incurrence was significant lower in the ACF cohort compared to PCF cohort (OR = 0.32; 95 % CI: [0.12–0.90]; p = 0.030).

**Table 3:** Association between catastrophic costs and baseline characteristics for the entire episode of TB

Baseline characteristics	Catastrophic cost incurrence^c^ N (%)^d^	Crude OR (95 %CI)	Adjusted^e^ OR (95 %CI)
Case finding model			
PCF^a^	13/44 (30)	1.00	1.00
ACF	8/52 (15)	0.43 (0.16–1.17)	0.17 (0.05–0.67)*
Sex			
Female^a^	6/35 (30)	1.00	1.00
Male	15/61 (25)	1.58 (0.55–4.52)	1.65 (0.49–5.53)
Age category^b^			
≤50 years^a^	9/51 (18)	1.00	1.00
>50 years	12/45 (27)	1.70 (0.64–4.51)	2.09 (0.67–6.52)
Education level			
Up to primary school only^a^	11/33 (33)	1.00	1.00
Secondary school and above	10/63 (16)	0.38 (0.14–1.02)	0.15 (0.04–0.60)*
Employment status			
Unemployed^a^	4/25 (16)	1.00	1.00
Employed	17/71 (24)	1.65 (0.50–5.49)	4.14 (0.83–20.76)
Social Health Insurance status			
Uninsured^a^	5/20 (25)	1.00	1.00
Insured	16/76 (21)	0.80 (0.25–2.53)	0.78 (0.19–3.23)

^a^Referent;

^b^Age>50 years (mean age for pooled sample);

^c^*N*=96;

^d^Denotes participants that incurred catastrophic costs over the total number of people in a given subgroup;

^e^Multivariate logistic regression incorporating all shown patient covariates (i.e., case finding model, sex, etc.) as exposure parameters for the model

* Statistically significant difference at 95 % confidence level

**Table 4:** Socioeconomic impact and catastrophic costs in patients diagnosed through ACF and PCF at individual treatment periods

Variables	Pre-treatment, N (%)		OR^d^ (95 % CI)	Intensive phase, N (%)		OR^d^ (95 % CI)	Continuation phase, N (%)		OR^d^ (95 % CI)	End of treatment, N (%)		OR^d^ (95 % CI)
	**ACF** **(*N* = 52)**	**PCF** **(*N* = 46)**		**ACF** **(*N* = 52)**	**PCF** **(*N* = 46)**		**ACF** **(*N* = 52)**	**PCF** **(*N* = 46)**		**ACF** **(*N* = 52)**	**PCF** **(*N* = 46)**	
Catastrophic costs^a^	8 (15)	13 (30)	0.43 (0.16–1.17)	8 (22)	17 (46)	0.32 (0.12–0.90)*	11 (22)	12 (27)	0.76 (0.30–1.93)	10 (20)	10 (22)	0.86 (0.32–2.29)
Unemployed	5 (10)	8 (17)	0.51 (0.15–1.67)	15 (29)	21 (46)	0.48 (0.21–1.11)	10 (19)	10 (22)	0.86 (0.32–2.29)	7 (13)	9 (20)	0.64 (0.22–1.88)
Poverty headcount^b^	11 (21)	8 (17)	1.27 (0.46–3.51)	22 (42)	19 (41)	1.04 (0.47–2.33)	12 (23)	10 (22)	1.08 (0.42–2.80)	11 (21)	9 (20)	1.10 (0.41–2.96)
Social exclusion	NA	NA	NA	21 (66)	11 (34)	2.16 (0.90–5.17)	19 (41)	27 (59)	0.41 (0.18–0.91)*	19 (45)	23 (55)	0.58 (0.26–1.29)
Loss of employment	NA	NA	NA	15 (29)	18 (39)	0.63 (0.27–1.47)	12 (23)	16 (35)	0.56 (0.23–1.36)	8 (15)	11 (24)	0.58 (0.21–1.59)
Financial impact^c^	NA	NA	NA	29 (56)	18 (39)	1.96 (0.87–4.39)	27 (52)	20 (43)	1.40 (0.63–3.11)	19 (37)	19 (41)	0.82 (0.36–1.84)
Coping strategies	NA	NA	NA	14 (27)	7 (15)	2.05 (0.75–5.64)	20 (38)	5 (11)	5.12 (1.73–15.14)*	15 (29)	11 (24)	1.29 (0.51–3.18)

^a^Catastrophic costs calculated according to the income reported in each period of analysis;

^b^Poverty line established by the World Bank = $1.90, International Dollar ($) applying purchase power parity (PPP), 2017 prices, conversion factor = $7,716.43;

^c^Patients indicating that they were poorer or much poorer as a result of their episode of TB;

^d^Univariate odds ratio accounting for autocorrelation using generalized estimation equation methods with working correlation structure obtained using quasi-likelihood information criteria

Participants in the ACF cohort further showed a significantly lower risk of social exclusion during the continuation phase (OR = 0.40; 95 % CI: [0.18–0.91]; p = 0.030). However, patients in the ACF cohort also had a significantly higher risk of resorting to financial coping mechanisms during the continuation phase (OR = 5.12; 95 % CI: [1.73–15.14]; p = 0.003).

## Discussion

We found that ACF significantly reduced catastrophic cost incurrence. Specifically, the study showed a lower proportion and odds of households experiencing catastrophic cost in the ACF cohort compared to the PCF cohort. This was potentially due the higher unemployment and income loss in the PCF cohort, which subsequently amplified the socio-protective effects of ACF. This is concordant with findings from other settings [12, 31].

Compared to the country [11] and region [32], the catastrophic cost prevalence in our study was lower. This was likely due to situating the study in HCMC, the economic center of Viet Nam [33, 34]. We measured substantially higher average monthly household incomes in our survey (US$483) than in the national patient cost survey (US$322) [11]. A survey in Indonesia observed a similar effect when dichotomizing catastrophic cost incurrence between poor and non-poor TB patients [35]. As catastrophic cost incurrence is defined on the basis of household income [36], the temporary loss of one source of income may have been compensated by the remaining household members in our setting. Therefore, it will be important to assess the productivity of the other household members, when evaluating vulnerability to catastrophic cost incurrence and optimizing eligibility for social protection mechanisms.

One critical yet underemphasized benefit of ACF is the ability to reach more marginalized populations and increase equity in TB care [31, 37]. Our study’s ACF activities evinced similar benefits as we reached more socioeconomically disadvantaged groups, who more commonly lacked basic essentials and access to social protection mechanisms. TB patients in the ACF cohort more commonly resorted to financial coping mechanisms to manage the socioeconomic burden of TB. As such, it was encouraging to observe that our ACF activities optimized the TB care pathway. ACF patients reported fewer health-seeking events before treatment and fewer hospitalizations than PCF patients. ACF patients also more frequently sought care directly with the NTP, while PCF patients tended to access several healthcare providers before finally reaching the NTP [38].

It may be that the ACF cohort was unable to afford other alternatives, and therefore were receptive to the CHW’s referral to the NTP. This is also supported by our finding that ACF did not significantly reduce the time between symptom onset and treatment initiation, i.e., diagnostic delays, as observed in other settings [39]. Particularly, the hypothesis is that ACF patients were significantly poorer and as such potentially delayed accessing healthcare altogether until approached and counseled by a CHW. Meanwhile, the PCF cohort visited private and non-NTP public providers at a higher rate prior to reaching the NTP. This optimization of the patient pathway is a key socio-protective effect of ACF that has also been documented elsewhere [40–42].

The reduction in health facility visits resulting from CHW referrals to the NTP optimized the patient pathway and likely helped to reduce costs among ACF patients. A systematic review of TB-related patient costs in sub-Saharan Africa shows that policies and programs improving access to healthcare has the potential to reduce pre-treatment costs, though the optimal modality for reducing costs to patients remains unknown [43]. ACF patients also reported significantly fewer costs from lost income. Past studies identified this category as a major source of economic burden for TB patients [11, 44]. A reason may be that the socio-protective effects of community-based ACF do not end after enrollment. Patient support provided by CHWs throughout treatment, especially during the intensive phase, can ameliorate economic burdens as well. This included collection and transport of sputum for follow-up testing and provision of counseling and psychosocial support. These services were offered free of charge thereby alleviating any associated costs for the patient. Our study also found that ACF patients incurred significantly lower direct medical costs during treatment. PCF patients reported more additional visits to private providers during the treatment phase (see Table 1), which resulted in higher costs from medical fees and consultations. While our health economic evaluation of these activities is ongoing, they comprise well-documented benefits of community-based care [45, 46].

It is important to note that within our study sample, the PCF cohort reported significantly higher pre-treatment individual and household incomes. However, once TB was diagnosed and treatment commenced we observed no significant differences between the PCF and ACF cohorts. This either suggests that TB had a greater impoverishing effect on PCF patients or could also mean that better socioeconomic conditions afforded the freedom to forego income-generating activities. This is supported by the higher rates of formal employment among PCF patients, who were more likely to benefit from statutory job protections, such as paid sick leave and protection from unlawful termination [47].

Through the longitudinal design of our study, we were able to observe a near-full recovery of household income loss and employment in both cohorts during the continuation phase. The recovery was likely a consequence of TB patients returning to work to recover from the loss in productivity experienced during the illness, whether it was related to directly observed treatment, TB-related disability or statutory leave during the intensive phase. A similar longitudinal survey conducted among ACF and PCF TB patients in Nepal did not show any income recovery during treatment. This discordance was possibly a result of Viet Nam’s stronger economy, which offered more employment options after the completion of TB treatment, a greater ability for TB patients to re-enter the job market, and to recover from the deleterious socioeconomic impact of TB.

The socioeconomic burden throughout individual treatment phases requires further investigation, but our findings that the burden was highest during the intensive phase may inform and optimize social protection mechanisms. As attention to social protection schemes, such as cash transfers, is increasing, [48] it may be worthwhile to systematically compare the effects of providing social support during the intensive phase only versus throughout the full treatment course. This approach has the potential to optimize resource allocation for social protection schemes.

Our study was subject to recall and social desirability bias. We believe the longitudinal design of the survey and audit of receipts mitigated the impact of these limitations, but also required a concerted effort to remind patients of the period in question when assessing costs, as well as time and income losses. Focusing solely on HCMC biased our data towards a higher socioeconomic class and limited the generalizability of our findings in the national and international context. As the study was conducted under programmatic conditions, patient follow-up was periodically affected by supply chain and program irregularities. Through close collaboration with the provincial and national TB control programs, we tried to minimize the impact of these irregularities.

## Conclusions

ACF can be effective in reaching vulnerable populations and mitigating the socioeconomic burden of TB. Programs should consider ACF a key strategy to support TB-affected families and achieve the WHO End TB Strategy goals. Nevertheless, as TB remains a catastrophic life event, social protection efforts must extend beyond ACF. Given resource limitations, it may be possible to focus these efforts on TB patients in the intensive phase of treatment so that more TB–affected families can receive support.

## Supplementary Information

**Additional file 1:**

## Data Availability

The data that support the findings of this study are available from the Viet Nam National TB Control Program and Pham Ngoc Thach Provincial TB Hospital, but restrictions apply to the availability of these data, which include programmatic clinical patient information, and so are not publicly available. Data are can be made available from the authors upon reasonable request and with permission of the Viet Nam National TB Control Program and Pham Ngoc Thach Provincial TB Hospital.

## Abbreviations

ACF: Active Case Finding
CHW: Community Health Worker
CI: Confidence Interval
CXR: Chest X-ray
DS-TB: Drug-Susceptible Tuberculosis
EP: Extra-pulmonary
GEE: Generalized Estimating Equation
HCMC: Ho Chi Minh City
MDR-TB: Multi-drug Resistant Tuberculosis
MPI(-TB): Multidimensional Poverty Index (for TB)
NTP: National TB Control Program
(a)OR: (adjusted) Odds Ratio
PCF: Passive Case Finding
SHI: Social Health Insurance
TB: Tuberculosis
WHO: World Health Organization
