# Supplementary material for: Socio-protective effects of active case finding on catastrophic costs from tuberculosis in Ho Chi Minh City, Viet Nam: a longitudinal patient cost survey
Source: BMC Health Serv Res. 2021 Oct 5;21:1051. doi: 10.1186/s12913-021-06984-2 (PMC8493691; doi:10.1186/s12913-021-06984-2)
Supplement: Supplementary file 1 — Additional file 1: [file 12913_2021_6984_MOESM1_ESM.docx]

# SUPPLEMENTAry MATERIAL

## Supplementary methods

*Multidimensional poverty index for TB*

We constructed a multidimensional poverty index for TB patient households in our study (MPI-TB) by applying the Alkire-Foster method^27^ and poverty dimensions outlined by the Government of Viet Nam^26^. These dimensions include health, education, social protection, living standards, and access to information/social participation. We assigned 16 indicators obtained from the WHO patient cost tool to construct the MPI-TB to these five dimension and weighted each dimension and indicator equally based on established methods (Table S1). We calculated the crude and adjusted MPI-TB based on the intensity of deprivation^27^, and estimated the proportion of TB patients under this study’s pre-defined threshold of 0.33. This threshold was based on the government-defined threshold^26^ that and identifies a poor household in terms of income poverty and multidimensional poverty.

Table S1. Adapted multidimensional poverty index for TB patient households

| **Dimensions** | **Indicators** | **Weights** |
| --- | --- | --- |
| Health | Not enrolled onto social health insurance | 1/15 |
|  | TB patient with co-morbid HIV/AIDS | 1/15 |
|  | Household had more than one TB patient | 1/15 |
| Education | TB patient did not attain upper secondary school or vocational training degrees | 1/10 |
|  | Head of household did not attain upper secondary school or vocational training degrees | 1/10 |
| Social protection | TB patient did not access government social protection schemes throughout treatment | 1/15 |
|  | TB had to take out at least one loan throughout treatment | 1/15 |
|  | TB had to sell at least one assets throughout treatment | 1/15 |
| Living standards | Household does not have access to the national electricity grid | 1/20 |
|  | Household does not have access to piped water | 1/20 |
|  | Household does not have access to hygienic latrine | 1/20 |
|  | Household does not have a motorbike | 1/20 |
| Information access/ social participation | Household does not have a television | 1/20 |
|  | Household does not have a computer | 1/20 |
|  | Household does not have a telephone | 1/20 |
|  | TB patient perceived stigma, social exclusion or the need for self-isolation due to the illness^a^ | 1/20 |

^a^Entailed any perceived segregation ranging from distancing to discrimination by neighbors, colleagues, friends and family members as well as the TB patients self-imposed isolation and hesitation to interact with household members.

## Supplementary results

A description of the living standards and household assets collected through the WHO patient cost survey tool and incorporated into the MPI-TB for TB is in Table S2. The largest number of deprivations among TB patients across both cohorts was in the education and social protection dimensions. These also contained the indicators on which we observed the largest gaps between ACF and PCF cohorts. In relation to each other, a significantly higher share of patients in the ACF cohort had no SHI (31% vs. 9%), lived in households whose head did not complete secondary school (76% vs. 49%), were unable to access formal social protection schemes throughout the episode of TB, (98% vs. 85%) and had no access to piped water (19% vs. 0%).

Table S2. TB patient characteristics in the ACF and PCF cohorts used for the calculation of the MPI-TB

|  | **ACF**  **(N= 52)** | **PCF**  **(N = 46)** | **All**  **(N = 98)** | **P-value^a^** |
| --- | --- | --- | --- | --- |
| **MPI-TB depravity indicators, N (%)** |  |  |  |  |
| No Social Health Insurance | 16 (31) | 4 (9) | 20 (20) | 0.007* |
| TB/HIV co-infection | 0 (0) | 3 (7) | 3 (3) | 0.061 |
| Multi-TB patient household | 10 (19) | 3 (7) | 13 (13) | 0.064 |
| No secondary school – TB patient | 24/34 (71) | 13/24 (54) | 37/58 (64) | 0.200 |
| No secondary school – Household head | 39/51 (76) | 21/43 (49) | 60/94 (64) | 0.005* |
| No social protection scheme accessed | 51 (98) | 39 (85) | 90 (92) | 0.016* |
| Loan taken | 11 (21) | 5 (11) | 16 (16) | 0.169 |
| Assets sold | 3 (6) | 1 (2) | 4 (4) | 0.369 |
| No electricity | 1 (2) | 0 (0) | 1 (1) | 0.344 |
| No piped water | 10 (19) | 0 (0) | 10 (10) | 0.002* |
| No flush toilet | 20 (39) | 11 (24) | 31 (32) | 0.122 |
| No motorbike | 3 (6) | 3 (6) | 6 (6) | 0.877 |
| No television | 4 (8) | 6 (13) | 10 (10) | 0.382 |
| No computer | 35 (67) | 23 (50) | 58 (59) | 0.082 |
| No telephone | 0 (0) | 1 (2) | 1 (1) | 0.285 |
| Social exclusion | 32 (62) | 25 (54) | 57 (58) | 0.471 |
| Adjusted MPI-TB headcount ratio, mean (SD)^2^ | 15.5 (21.8) | 7.1 (15.9) | 11.6 (19.6) | 0.033* |

^a^Chi-squared test.

* Statistically significant difference at 95% confidence level.

Figure S1 shows the median (IQR) and mean (95% CI) as well as minimum and maximum proportion of total costs as a percentage of pre-treatment household income in the ACF and PCF cohorts. The median proportions were 3.8% (IQR: 2.4%–14.7%) and 23.5% (IQR: 5.0%–23.9%), respectively. The mean proportion was 10.5% (95% CI: [6.2%, 14.7%]) in the ACF cohort and 23.5% (95% CI: [9.0%, 38.0%]) in the PCF cohort.

Figure S1. Total costs incurred as a proportion of pre-treatment household income.


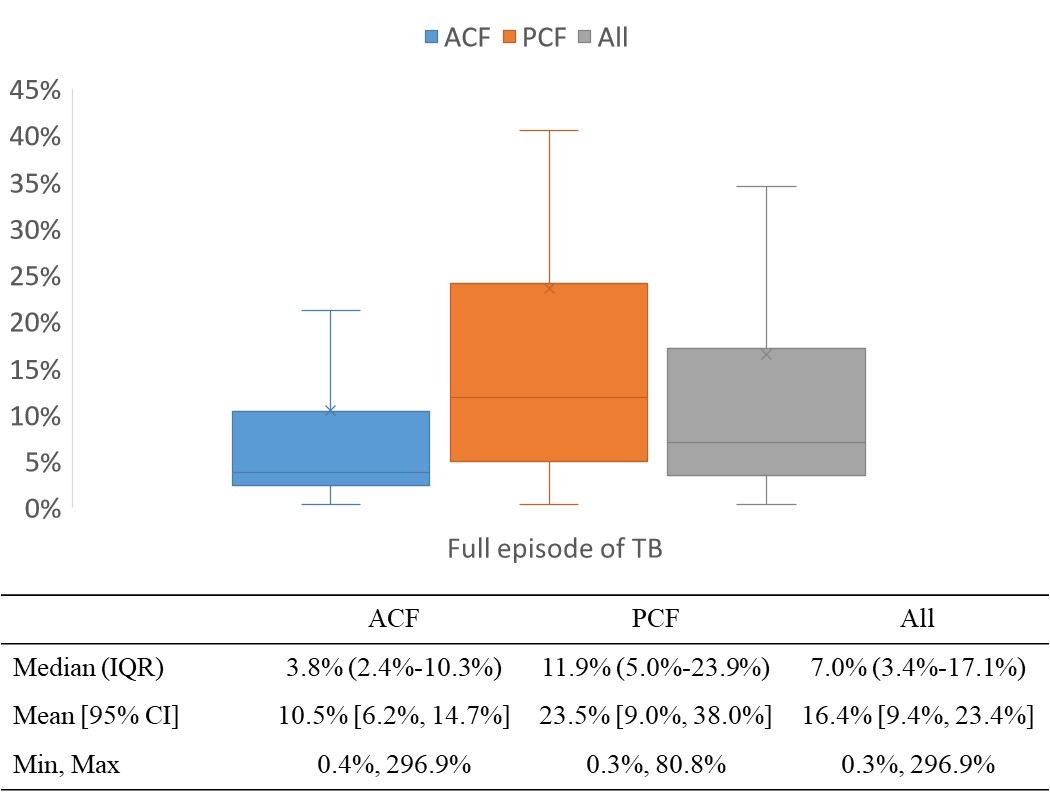


## STROBE Statement

Table S3. Completed STROBE checklist — cross-sectional studies

|  | Item No | Recommendation | Page No |
| --- | --- | --- | --- |
| **Title and abstract** | 1 | (*a*) Indicate the study’s design with a commonly used term in the title or the abstract | 1, 4 |
|  |  | (*b*) Provide in the abstract an informative and balanced summary of what was done and what was found | 4 |
| Introduction | | | |
| Background/rationale | 2 | Explain the scientific background and rationale for the investigation being reported | 5 |
| Objectives | 3 | State specific objectives, including any prespecified hypotheses | 5 |
| Methods | | | |
| Study design | 4 | Present key elements of study design early in the paper | 5-6 |
| Setting | 5 | Describe the setting, locations, and relevant dates, including periods of recruitment, exposure, follow-up, and data collection | 6 |
| Participants | 6 | (*a*) Give the eligibility criteria, and the sources and methods of selection of participants | 6 |
| Variables | 7 | Clearly define all outcomes, exposures, predictors, potential confounders, and effect modifiers. Give diagnostic criteria, if applicable | 6-7 |
| Data sources/ measurement | 8* | For each variable of interest, give sources of data and details of methods of assessment (measurement). Describe comparability of assessment methods if there is more than one group | 6-7 |
| Bias | 9 | Describe any efforts to address potential sources of bias | 6 |
| Study size | 10 | Explain how the study size was arrived at | 6 |
| Quantitative variables | 11 | Explain how quantitative variables were handled in the analyses. If applicable, describe which groupings were chosen and why | 6-8 |
| Statistical methods | 12 | (*a*) Describe all statistical methods, including those used to control for confounding | 7-8 |
|  |  | (*b*) Describe any methods used to examine subgroups and interactions | 7-8 |
|  |  | (*c*) Explain how missing data were addressed | 8 |
|  |  | (*d*) If applicable, describe analytical methods taking account of sampling strategy | 8 |
|  |  | (*e*) Describe any sensitivity analyses | n/a |
| Results | | | |
| Participants | 13* | (a) Report numbers of individuals at each stage of study—eg numbers potentially eligible, examined for eligibility, confirmed eligible, included in the study, completing follow-up, and analysed | 8 |
|  |  | (b) Give reasons for non-participation at each stage | 8 |
|  |  | (c) Consider use of a flow diagram | eFig 2 |
| Descriptive data | 14* | (a) Give characteristics of study participants (eg demographic, clinical, social) and information on exposures and potential confounders | 8 |
|  |  | (b) Indicate number of participants with missing data for each variable of interest | 17-20 |
| Outcome data | 15* | Report numbers of outcome events or summary measures | 8-10 |
| Main results | 16 | (*a*) Give unadjusted estimates and, if applicable, confounder-adjusted estimates and their precision (eg, 95% confidence interval). Make clear which confounders were adjusted for and why they were included | 9-10 |
|  |  | (*b*) Report category boundaries when continuous variables were categorized | n/a |
|  |  | (*c*) If relevant, consider translating estimates of relative risk into absolute risk for a meaningful time period | n/a |
| Other analyses | 17 | Report other analyses done—eg analyses of subgroups and interactions, and sensitivity analyses | 9-10 |
| Discussion | | | |
| Key results | 18 | Summarise key results with reference to study objectives | 10 |
| Limitations | 19 | Discuss limitations of the study, taking into account sources of potential bias or imprecision. Discuss both direction and magnitude of any potential bias | 12 |
| Interpretation | 20 | Give a cautious overall interpretation of results considering objectives, limitations, multiplicity of analyses, results from similar studies, and other relevant evidence | 12 |
| Generalisability | 21 | Discuss the generalisability (external validity) of the study results | 12 |
| Other information | | | |
| Funding | 22 | Give the source of funding and the role of the funders for the present study and, if applicable, for the original study on which the present article is based | 2 |
